# Factors Associated with Underweight among Children of Former-Kamaiyas in Nepal

**DOI:** 10.3389/fpubh.2015.00011

**Published:** 2015-01-29

**Authors:** Resham B. Khatri, Shiva R. Mishra, Vishnu Khanal, Bishnu Choulagai

**Affiliations:** ^1^Saving Newborn Lives Program, Save the Children, Kathmandu, Nepal; ^2^Monamohan Memorial Institute of Health Science, Kathmandu, Nepal; ^3^Nepal Development Society, Kathmandu, Nepal; ^4^School of Public Health, Curtin University, Perth, WA, Australia; ^5^Department of Internal Medicine and Clinical Nutrition, Institute of Medicine, University of Gothenburg, Gothenburg, Sweden; ^6^Department of Community Medicine and Public Health, Institute of Medicine, Tribhuvan University, Kathmandu, Nepal

**Keywords:** factors, Nepal, under five children, undernutrition, underweight

## Abstract

**Background:** Bonded labor was a tradition in Nepal since the 16th century. In 2002, the Government of Nepal freed Kamaiyas and gave the newly freed individuals support for basic living. Many children of former-Kamaiyas live below subsistence level and are vulnerable to undernutrition. The aim of this study was to identify the factors associated with underweight among the children of former-Kamaiyas.

**Methods:** We conducted the community based cross-sectional study from June to December, 2012. Face-to-face interviews were conducted using semi-structured questionnaires with randomly selected mothers of 280 children under 5 years of age from former-Kamaiya families residing in Banke district. We also measured the weight and height of the children. Undernutrition was defined according to the World Health Organization child growth standards. Factors associated with underweight were examined using a Chi-square test followed by multiple logistic regression.

**Results:** Out of 280 children, 116 (41.4%) were underweight (≤2 SD weight-for-age), 156 (55.7%) were stunted (≤2 SD height-for-age), and 52 (18.6%) were wasted (≤2 SD weight-for-height). Females were more likely to be underweight than males [adjusted odds ratio (aOR) = 1.696, 95% confidence interval (CI) = 1.026–2.804]. Children were less likely to be underweight if they were having daily bath (aOR = 0.532; 95% CI = 0.314–0.899) or if their mothers were ≥24 years of age (aOR = 0.440; 95% CI = 0.266–0.727).

**Conclusion:** The proportion of underweight, stunting, and wasting was more than the national average among the children of former-Kamaiyas. Female children were more likely to be underweight whereas children who were being bathed daily and with mothers whose age was ≥24 years were less likely to be underweight.

## Introduction

The practice of bonded labor was centuries-old in Nepal. According to an old estimate, there were between 300,000 to 2,000,000 agricultural bonded laborers in 1993, mainly concentrated in the Far-Western Hill and Terai districts (1–3). There were mainly two forms of traditional agricultural bonded labor system, namely Haliya and Kamaiya (1, 2). The Kamaiya system was mostly prevalent in the Tharu community of western Nepal (4), whereas Haliya system was prevalent practice in various castes and ethnic groups across Nepal.

Tharu ethnic group constituted 6.6% (1,737,470) of the total population of Nepal in 2011. They are inhabitants of the Central and Western Terai (plain) regions, as well as the inner low lands (5). Poor Tharu people would make verbal agreements extending up to a year with rich landlords from other castes but they usually became indebted whilst fulfilling their families’ basic needs, thus creating a multi-generational cycle of bonded laborers (1). Such laborers are called Kamaiyas and this system was primarily present in five western districts, namely Dang, Banke, Bardiya, Kailali, and Kanchanpur (1, 2).

The Government of Nepal outlawed the practice of all forms of bonded labor by introducing the “Bonded Laborers (Prohibition) Act 2002,” which freed the laborers in July, 2002. As a result, the 18,400 former-Kamaiya families mostly settled in Dang, Banke, and Bardiya. After more than one decade of their freedom, however, the former-Kamaiyas are among the most socio-economically disadvantaged groups (6). When the Government of Nepal declared Kamaiyas free, thousands were evicted from their masters’ homes and were deprived of access to land and work. Inadequate government support led many to live in the fields with no means of livelihood. Given that most of the former-Kamaiyas families are living under extreme poverty, with inadequate means of livelihood, and lack of food supplements, their children are vulnerable to undernutrition and other poor health condition.

Children who are underweight have low weight for their age, and this reflects a combination of chronic and acute malnutrition (wasting). Stunting reflects chronic nutritional deficiency (7). According to the Nepal demographic and health survey (NDHS) 2011, the prevalence of underweight, stunting, and wasting in children under 5 years of age was 40.3, 56.0, and 12.50%, respectively, in the lowest wealth quintile compared to 10.0, 25.8, and 7.4%, respectively, in the highest wealth quintile. In the Mid-Western region, the respective prevalence was 36.9, 50.3, and 11.3% (8). A study on the Chepang community, a marginalized ethnic group, showed that 37.3% of children were underweight (9). The undernutrition status of children in other marginalized communities, such as the children of former-Kamaiyas have not been adequately investigated. Therefore, the objective of this study was to identify the factors associated with underweight among children under the age of five.

## Materials and Methods

### Study setting and participants

The study was conducted in a former-Kamaiyas settlement in Kohalpur village development committee (VDC) of Banke district in the Mid-Western Development Region of Nepal. Kohalpur VDC has 8173 households with 36,019 people (male:female = 0.94:1) (5). About 20% of the population in the VDC is illiterate and 68.0% of former-Kamaiyas are illiterate. Almost one-fifth of the population in the VDC are Tharu including former-Kamaiyas (5). The majority of the population depends on livestock (52.9%) and manual labor work (21.9%) for their livelihood. In 2005, the Government of Nepal settled 902 households of former-Kamaiyas in three settlement areas of this VDC. The government provided 170–2028 m^2^ of land for shelter per household (10).

### Study design and sampling

We conducted a community based cross-sectional study from June to December, 2012. Face-to-face interviews were conducted with the mothers of children under the age of five using structured questionnaires. The list of children of former-Kamaiyas under the age of five was obtained from the local sub-health post. We calculated the sample size using the formula (11) (*n*) = *Z*^2^*pq*/*d*^2^ where *p* is the national prevalence of underweight among children under 5 years of age in the lowest wealth quintile = 40% (8), *q* = 1 − *p* = 60%, *d* is the allowable error = 6% (11) and assuming a 10% non-response rate. Thus obtained sample size of 282 was selected from 902 households, using a systematic random sampling. The weight and height of children were measured. If there was more than one child in the family, the youngest child was included in the study. However, two mothers did not respond, so a total of 280 mothers were included in the analysis.

### Materials and data collection

A structured questionnaire was adapted from the NDHS 2011 (8) and similar studies conducted in Nepal (9, 12). The questionnaire was pre-tested in Ward 2 of Rajhena VDC in Banke district to test cultural adaptability. We conducted 2-day training for five enumerators on interview techniques, data collection, and anthropometric measurements (weight and height). Recumbent length was measured for children 0–23 months of age and standing height was measured for children 24 months and older (13). Calibration of the instruments was carried out prior to record height and weight. Height was measured to the nearest to 1 cm. Weight was measured using a standardized calibrated Salter scale, which could detect mass to the nearest to 0.05 kg. Two measurements were taken and averaged.

### Measurement of variables

Outcome variable of this study was underweight. According to the World Health Organization (WHO), underweight, stunting, and wasting are the Z score of weight-for-age (WAZ), height-for-age (HAZ), and weight-for-height (WHZ), respectively, defined by ≤2 SD from the respective reference median (14).

Independent variables were demographic characteristics, breastfeeding history (early initiation of breastfeeding within 1 h and exclusive breastfeeding), pre-lacteal feeding (feeding the child liquids other than the mother’s milk prior to initiating lactation), 24-h recall of dietary diversity in meals (cereals, legumes, dairy, meat, fish, vitamins, and fruits/vegetables), and health and sanitation factors (Vitamin A and Albendazole tablet consumption in the past 6 months, growth monitoring, hand washing of children before eating, and stool disposal).

### Data analysis

We entered the data in EpiData 3.1. Anthropometric scores were generated using the software, ENA for SMART. Data entered into EpiData and ENA for SMART were exported to SPSS 19.0 for further analysis. The undernutrition status and nutritional practices were reported as proportions. Chi-square tests of association between underweight and independent variables were examined. Variables which were significant at the 25% level in the Chi-square test were further investigated in multiple logistic regression (15) using a stepwise backward elimination procedure. *P* values <0.05 were considered statistically significant in final multiple regression analysis.

### Ethical considerations

Ethics approval was obtained from the Institutional Review Board at the Department of Community Medicine and Public Health, Institute of Medicine, Tribhuvan University, Kathmandu, Nepal. Written approval was also given by the District Public Health Office, Banke and Kohalpur sub-health post. Participants also gave verbal informed consent.

## Results

### Characteristics of participants

Among the 280 mothers interviewed, the mean age was 24.0 (SD 4.8) years. The majority (56.8%) of mothers could not read and write at all. The average size of households was 5.1 (SD 1.8). The birth interval between the last child and the preceding child was >24 months for the majority (84.4%, *n* = 154) of mothers. Only 12.5% mothers had monthly earnings. The majority (53.9%) were living in the settlement for ≥7 years. The daily expenditure on food in most of the households (61.1%) was <200 Nepalese Rupees (≤2 USD). About 28% of mothers fed their children three or more types of food varieties as part of their typical daily meal (Table 1).

**Table 1:** **Characteristics of respondent mothers (*n* = 280)**.

Characteristics	Number (%)	Underweight^a^ (%)	*P* value
Age of mother; mean: 24.5 (SD: 4.8) years			***P* = 0.03**
<24 years	157 (56.1)	53 (33.8)	
≥24 years	123 (43.9)	63 (51.2)	
Family size; mean: 5.0 (SD: 1.8)			*P* = 0.34
2–5	187 (66.8)	90 (43.1)	
6–10	93 (33.2)	26 (36.6)	
Years of schooling of mother; median: <1 [IQR: 0–6] years			*P* = 0.37
<1 years (no schooling-illiterate)	159 (56.8)	73 (54.9)	
1–8 years	50 (17.9)	14 (34)	
≥9 years	71(25.4)	25 (35.21)	
Monthly earning			*P* = 0.85
No	245 (87.5)	101 (41.1)	
Yes	35 (12.5)	15 (42.9)	
Duration of living in the settlement; median: 7 [IQR: 4–9] years			***P* = 0.11**
2–7 years	129 (46.1)	60 (46.3)	
8–11 years	151(53.9)	56 (37.1)	
Expenditure on food in the past 24 h; median: 2 [IQR: 1–3] US Dolor			***P* = 0.03**
0–2 US Dolor	171(61.1)	62 (36.3)	
3–10 US Dolor	109 (38.9)	54 (49.3)	
Food diversity (*n* = 257)			*P* = 0.36
≤3 Food stuffs	185 (72.0)	81 (43.8)	
>3 Food stuffs	72 (28.0)	27 (37.5)	
Age of child; median: 27 [IQR: 13–40] months			*P* = 0.55
0–11	58 (20.7)	20 (34.5)	
12–23	53 (26.1)	33 (45.2)	
24–35	46 (16.4)	20 (43.5)	
36–47	62 (22.0)	23 (37.1)	
48–59	41 (14.6)	20 (48.8)	
Sex of child			***P* = 0.09**
Male	150 (53.6)	69 (46.0)	
Female	130 (46.4)	47 (36.2)	
Birth interval of child (*n* = 154); mean: 44.6 (SD: 21.2)			*P* = 0.87
10–24 months	24 (15.6)	9 (37.5)	
24–99 months	130 (84.4)	51 (39.2)	

*P value; Chi-square test of association*,

*^a^percentages indicate row percentage*.

*IQR, inter quartertile range*.

### Child feeding practices

Eight percent of children did not receive colostrum. Only 4.3% children received pre-lacteal feeds. About 82.9% mothers started breastfeeding their newborns within 1 h of birth. More than half (53.2%) of the mothers continued breastfeeding until the children reached 32 months of age. Similarly, 80% of mothers exclusively breast fed their children for 6 months. Less than half (43.2%) of the mothers practiced child feeding on demand (Table 2).

**Table 2:** **Child feeding practices (*n* = 280)**.

Characteristics	Number (%)	Underweight^a^ (%)	*P* value
Colostrum feeding practices			*P* = 0.37
Fed to newborn	259 (92.5)	104 (40.2)	
Discarded	21 (7.5)	12 (57.1)	
Practices of prelacteal feeding			*P* = 0.41
No	268 (95.8)	111 (41.0)	
Yes	12 (4.3)	4 (33.3)	
Time of early breastfeeding; median: 0.5 [IQR: 0.5–1] (h)			***P* = 0.07**
<1 h	232 (82.9)	89 (38.4)	
1–24 h	36 (12.85)	20 (55.6)	
≥24 h	12 (4.28)	7 (58.3)	
Feeding status of children			*P* = 0.77
Breastmilk only	23 (8.2)	8 (34.8)	
Breastmilk and solid meal	178 (63.6)	74 (41.6)	
Solid meal	79 (28.2)	34 (43.0)	
Termination of breastfeeding (*n* = 79); mean: 32 (SD: 4.9) months			*P* = 0.34
≥21–32 months	37 (46.8)	18 (48.6)	
33–40 months	42 (53.2)	16 (43.0)	
Duration of exclusive breastfeeding (*n* = 257); mean: 6.1(SD: 0.9) months			***P* = 0.14**
1–5 months	19 (7.4)	5 (26.3)	
6–9 months	238 (92.6)	103 (43.3)	
Feeding practices during illness			***P* = 0.22**
As per interests of child	121 (43.2)	55 (45.5)	
More than usual	97 (34.6)	32 (33.0)	
As usual	36 (12.9)	17 (42.2)	
Less than usual	26 (9.3)	12 (46.2)	

*P value; Chi-square test of association*,

*^a^percentages indicate row percentage*.

*IQR, inter quartertile range*.

### Health and sanitation practices

Almost 65.7% of the children were not monitored for growth. A vast majority (91.1%) of the children received Vitamin A and Albendazole (89.0%) in the last 6 months. About 68.2% of the children washed their hands with soap and water before eating (Table 3).

**Table 3:** **Health and sanitation practices (*n* = 280)**.

Characteristics	Number (%)	Underweight^a^ (%)	*P* value
Growth monitoring in past 6 months			***P* = 0.14**
No	184 (65.7)	82 (44.6)	
Yes	96 (34.3)	34 (35.4)	
Vitamin A capsule supplementation (6–59 months) (*n* = 236)			*P* = 0.46
No	21 (8.9)	6 (28.7)	
Yes	215 (91.1)	96 (44.7)	
Albendazole distributed children aged 1–5 years (*n* = 191)			***P* = 0.16**
No	21 (11)	9 (42.8)	
Yes	170 (89.0)	73 (42.9)	
Hand washing with detergents in the past 24 h (*n* = 220)			*P* = 0.41
No	89 (31.8)	40 (44.9)	
Yes	131 (68.2)	76 (39.8)	
Child bathing practices in the last 7 days			***P* = 0.02**
Bathing in alternative days	87 (31.1)	45 (51.7)	
Daily bathing	193 (68.9)	71 (36.8)	
Disposal of children stool in the past 24 h			***P* = 0.07**
Open defecation	134 (47.9)	64 (47.8)	
Sanitary latrine	75 (26.8)	24 (32.0)	
Pit latrine	71 (25.4)	28 (39.4)	

*P value; Chi-square test of association*,

*^a^percentages indicate row percentage*.

*IQR, inter quartertile range*.

### Undernutrition status of children

Of the 280 children, 116 (41.4%) were underweight, 156 (55.7%) were stunted, and 52 (18.6%) were wasted (Table 4).

**Table 4:** **Undernutrition status of children (*n* = 280)**.

Undernutrition status	Number [%; 95% CI]	NDHS 2011	
		National (%)	Mid-Western Terai region (%)
Underweight	116 [41.4; 35.7–47.8]	29	32.1
Stunting	156 [55.7; 50–61.8]	41	43.5
Wasting	52 [18.6; 13.6–23.2]	11	7.5

### Factors associated with underweight

A number of independent variables were associated with being underweight in Chi-square test of association. But in multiple logistic regression, only three variables were significantly associated. Female children had higher odds of being underweight compared to their male counterparts [adjusted odds ratio (aOR) = 1.696; 95% confidence interval (CI) = 1.026–2.804]. On the other hand, children who were bathed daily had a lower likelihood of being underweight (aOR = 0.532; 95% CI = 0.314–0.899). Children whose mothers were ≥24 years of age were less likely to be underweight (aOR = 0.440; 95% CI = 0.266–0.727) (Table 5).

**Table 5:** **Factors associated with underweight**.

Characteristics	OR (95%CI)	*P* value	aOR (95%CI)	*P* value
Sex of child		*P* = 0.09		*P* = 0.04
Male	1		1	
Female	1.504 (0.930–2.433)		1.696 (1.026–2.804)	
Child bathing practices in the last 7 days		*P* = 0.02		*P* = 0.02
Bathed in alternative days	1		1	
Bathed daily	0.543 (0.325–0.907)		0.532 (0.314–0.899)	
Age of mothers		*P* = 0.03		*P* = 0.01
<24 years	1		1	
≥24 years	0.485 (0.299–0.788)		0.440 (0.266–0.727)	

*OR, odds ratio, aOR, adjusted odds ratio, CI, confidence interval*.

## Discussion

The proportion of underweight, stunting, and wasting was 41.4, 55.7, and 18.6%, respectively. The proportion of undernutrition in this community was higher than the national average, and also higher than in the Mid-Western Terai districts of Nepal (8). This proportion is higher than other studies conducted in countries like Kenya, Serbia, and India (8, 13, 16, 17). The high proportion of undernutrition might be due to the overall poor socio-economic status of the study population. In Nepal, only a third of children receive the recommended amount of food (18). The poor feeding practices in this socially marginalized community may have contributed to higher undernutrition among children. It is an emergency situation in this community, which calls for urgent interventions (19).

More than 82% of mothers were found to initiate breast feeding within 1 h of childbirth. NDHS 2011 and the study conducted in Pokhara, Nepal, reported breastfeeding within 1 h of child birth at 44.5 and 43.5%, respectively (8, 20). In our setting, such a high proportion of early initiation might be due to the cultural practice of initiating breastfeeding immediately after birth. The lower socio-economic status of this study group may be another factor that accounts for a high breastfeeding initiation rate as they may be unable to buy expensive pre-lacteal feeds (18). Likewise, the duration of exclusive breastfeeding was 6.1 months, which was higher than the national average (4.2 months) (8). However, this finding subjects to limitations of having long recall period. The current nutrition program of the Ministry of Health and Population of Nepal promotes exclusive breastfeeding up to the age of 6 months. Nepal’s Breast Milk Substitute Act (1992) promotes and protects breastfeeding and regulates the unauthorized or unsolicited sale and distribution of breast milk substitutes (21). Introducing breast milk substitutes to infants before 6 months can contribute to breastfeeding failure and infections.

Our study found that only 4% of mothers had provided pre-lacteal feeds to their children. Pre-lacteal feeding practices are common in Nepal as it is culturally accepted in some communities (18, 22). Previous studies conducted in Nepal have revealed that the prevalence of pre-lacteal feeding ranges from 14 to 39% (18, 21, 22). The Government of Nepal recommends colostrum feeding practices. The practice of providing pre-lacteal feeds is discouraged because it limits the frequency of suckling by the infant and exposes the baby to the risk of infection (8).

We found that female children were more likely to be underweight. However, the likelihood of underweight was found lower in children whose mothers were ≥24 years of age and those children who were bathed daily. A study from Ethiopia reported that male children were more likely to be underweight (23). Similar studies from other settings reported that the sex of the child was associated with the risk of undernutrition (17, 24). The frequency of bathing children may be taken as a proxy for general hygienic practices. The association between hygienic conditions and childhood diarrhea is well established (25). Our current findings support the idea that mothers who practice better hygiene by bathing their children frequently were less likely to be undernourished. Studies conducted in India and Bangladesh reported that children whose mothers were <20 years, were more likelihood to be undernourished (24, 26). Older mothers may be more able to care for their children. However, we did not examine other confounding factors such as number of illnesses the children suffered or quality of food and seasonal variation in such foods. Therefore, further research may be needed to include these variables.

This is the first study to explore the nutritional status among the children of former-Kamaiya families in Nepal. However, this study has a number of limitations. First, this was a cross-sectional study conducted in a small sample; the study might not be representative of all similar populations in Nepal. Recall bias may be a concern in nutritional practices of the mothers. Further research is needed to explore the ways to minimize undernutrition using cost-effective and sustainable technology.

## Conclusion

The proportion of undernutrition among the children of former-Kamaiya families was higher than the national average. Female children were more likely to be underweight. Children who were being bathed daily and born to mother’s ≥24 years of age were less likely to be underweight.

## Conflict of Interest Statement

We declare that we do not have competing interest. First author presented the abstract of this paper at the third national nutrition symposium held in Kathmandu on 18–20 November 2014.
